# Global Comparison and Future Trends of Major Food Proteins: Can Shellfish Contribute to Sustainable Food Security?

**DOI:** 10.3390/foods14132205

**Published:** 2025-06-23

**Authors:** Elena Tamburini, David Moore, Giuseppe Castaldelli

**Affiliations:** 1 Department of Environmental and Prevention Sciences, University of Ferrara, 44121 Ferrara, Italy; ctg@unife.it; 2 School of Biological Sciences, Faculty of Biology, Medicine and Health, University of Manchester, Manchester M13 9PT, UK; david@davidmoore.org.uk

**Keywords:** food security, climate change, shellfish, bivalve mollusks, terrestrial livestock, alternative protein

## Abstract

Food security and environmental quality related to food production are global issues that need urgent solutions. Proteins are crucial for diets, and demand is growing for innovative and more environmentally sustainable sources of protein, like vegetables, microorganisms, and insects, and lab-grown food that can meet nutritional and environmental goals. This study analyzes a time series to assess the sustainability of different protein sources by evaluating their effects on emissions of greenhouse gases and the use of agricultural land while accounting for the carbon sink potential across the supply chain. The study also explores future trends in global protein sources, emphasizing shellfish as a key to achieving food security from both nutritional and environmental perspectives. By reviewing terrestrial livestock, farmed seafood, vegetal proteins, and alternative sources like insects and cultured cells, the study assesses sustainability, food security potential, and challenges from nutritional, environmental, and consumer viewpoints. We conclude that shellfish aquaculture, particularly oysters, mussels, clams, and scallops, has significant potential in enhancing food security, fostering sustainable protein consumption, reducing land use, and contributing to climate change mitigation by sequestering significant amounts of atmospheric carbon.

## 1. Introduction

The latest United Nations report, which combines the mortality, fertility, and migration scenarios for 195 countries, indicates that the total world population, currently estimated to be 7.6 billion, is forecast to increase to 9.8 billion in 2050 and reach 11.2 billion in 2100 [1]. Moreover, the extent of the middle and rich classes, especially in Asian countries, has undergone rapid expansion, with a considerable impact on consumption patterns and for higher-value processed food [2]. With the rise in living standards, people, especially in urban areas, tend to move up in the food chain toward more energy-dense diets that include more animal products [3].

Aggregated demand for animal proteins is predicted to surpass that of all other foods by 2100, driven primarily by the South and South East Asia and Sub-Saharan Africa regions, whereas consumption in OECD (Organization for the Economic Cooperation and Development) countries will remain virtually unchanged, due to slow population growth and environmental concerns [4]. Similarly, the consumption of vegetal sources of protein, such as pulses, roots, tubers, and cereals, which continue to dominate the protein supply per capita, is expected to increase in the near future [5]. In fact, the animal-to-vegetable-protein ratio, which ranged from 0.29 in Africa to 1.08 in Europe in 2017 [6], is projected to nearly double in Africa by 2030 while remaining constant at 1.09 in Europe [7]. The market for so-called alternative proteins, such as plant-based, insect-based, cultured meat, and mycoprotein, is expected to grow in developed countries at a compounded average rate of 6% per year [8].

The existing challenges to world food production are predicted to be exacerbated by climate change, as increased atmospheric CO_2_ concentrations diminish the nutritional value of grains and legumes, impacting especially essential nutrients such as zinc and iron [9]. Meanwhile, the expanding global population is becoming wealthier, and rising incomes are driving greater consumption of resource-intensive foods, particularly those that are sources of proteins [10]. According to published estimates, the world will need to produce more food in the next 35 years than it has in all of human history, given projected population growth and the continued evolution of dietary preferences [11]. However, current dietary protein production and consumption are depleting resources, degrading the environment, and fueling chronic diseases [12]. A critical challenge lies in redesigning the food system to ensure it is healthy, sustainable, and resilient to climate change, thereby supporting the achievement of both the Sustainable Development Goals and the Paris Agreement [13]. These human and environmental impacts light up intense debate on how to shift away from resource-intensive animal-based proteins, while food security remains a complex and pressing challenge [14]. Notably, the environmental impact of agricultural protein production has already surpassed established planetary sustainability thresholds, including those related to climate change, land use change, biogeochemical fluxes, and biodiversity [15]. Agriculture, including livestock, currently contributes approximately 20% of anthropogenic greenhouse gas emissions and accounts for roughly 70% of global freshwater withdrawals, with its long-term sustainability increasingly jeopardized by prevailing trends [16].

On the other hand, there is much concern about future fish consumption based on expected population growth as well as due to the large number of people still suffering from undernourishment. At the same time, there may be limits to the potential for expanded production from wild-catch fisheries and intensive aquaculture because they are both not immune to environmental concerns, like overfishing or the release of fish feed and pharmaceuticals into the water [17]. In this context, bivalve aquaculture (i.e., clam, oyster, mussel, and scallop) could represent a key option to meet food security objectives from both nutritional and environmental perspectives while preserving low-impacts production, supporting land use reduction, and contributing to climate change mitigation via shell biocalcification [18]. The production is rapidly expanding, already accounting for over 15% of the animal protein consumption of approximately 1.5 billion people [19].

A substantial body of literature examines the environmental impact of producing various animal protein sources using the life cycle assessment (LCA) framework, including analyses of their respective nutrient values [20]. Life cycle assessment (LCA) is a suitable methodology for evaluating the potential impacts of protein production across the entire production cycle. LCA includes the extraction and processing of raw materials, manufacturing, transportation and distribution, usage, reuse, and maintenance, as well as recycling and waste management [21].

This critical review aims to investigate future global trends and environmental impacts of the main food protein. We have extended the selection to cover all principal protein sources for which data are available, including terrestrial livestock, farmed seafood, vegetal proteins, and alternative protein sources, like insect protein and cultured meat, with a focus on bivalve mollusks.

## 2. Materials and Methods

### 2.1. Data Collection Method

This study is a traditional systematic and critical review based on a mixed approach, merging literature data and data collection from public databases [22].

Our study integrates well with the mixed method, which seeks a more complete understanding through the integration of qualitative and quantitative research. Based on a reasoned and informed review of published research since 2000, the key methodological features have been collected in order to increase understanding. We have extracted live weight mass and raw production data in the time interval of 1990–2020 from the FAO database, the Food and Agriculture Statistics of the FAO (FAOStat Trade Data) [23], and FishStaJ 4.02.06, which collects global fisheries and aquaculture production data, as well as the daily protein supply per capita—the current one and that expected in 2050. Using these official FAO data, we selected the first three species in terms of production mass at the global level for protein categories of terrestrial animal, vegetable, and marine aquaculture protein sources. Specifically, we have selected poultry, pork, and beef as animal meats; whole wheat flour, soybeans, and peas like vegetable crops; milkfish (*Chanos chanos*), Atlantic salmon (*Salmo salar)* and rainbow trout (*Oncorhynchus mykiss*) like farmed fish; and farmed crustaceans, mainly white leg shrimp (*Penaeus vannamei*). We have also included insects and cultured meat as examples of innovative and alternative protein sources. The FAO lists only 79 marine bivalve mollusks farmed out of the approximately 8000 species grouped into four main groups of clams, oysters, scallops, and mussels. The latest available data summary from the FAO (2024) indicates that the production of bivalve mollusks in global aquaculture represents approximately 17 million tons per year. In this study, oysters, clams, and mussels have been included.

A total of 15 sources of food protein were included in this study. World productions data by species, current and forecast, have been obtained from the FAO database, whereas the consumption forecast and nutritional information are from literature (Table 1). All protein content, byproduct/edible weight ratio, Feed Conversion Ratio (FCR), and Nutrient Rich Food (NRF_9.3_) scores were expressed in live or fresh products. The 9.3 is a subscript that considers nine nutrients that are encouraged in the diet-protein (vitamins A, C, and D, calcium, fiber, potassium, protein, magnesium, and iron) and three nutrients that should be limited (added sugars, saturated fat, and sodium) [24,25]. FCR measures nutrient retention in terms of the weight of the feed fed to an animal over its lifetime divided by the live weight gained [26]. The standard deviation for protein content was indicated as that reported in the literature we accessed for each reported value.

### 2.2. Production-Related GHG Emissions, Land Use, and Carbon Capture Capacity

The data presented in Table 2 for the selected protein sources were derived from the scientific literature published since 2000. Several studies and reviews based on LCA were found, especially for terrestrial animal proteins and seafood. The main effort was to verify the system boundaries and functional units, as in some cases, the environmental impact calculations included a cradle-to-plate analysis of the CO_2_ eq. emissions that were calculated on the average cooked portion. We focused on the cradle-to-gate studies, up to the end of production (the gate of the production site) and up to 1 kg of live weight for animals and 1 kg of fresh product for vegetables. Unfortunately, it was more difficult to find studies on insects and cultured meat. According to the LCA methodology, one of the impact categories resulting from the analysis is the use of land, which declares the impact of managing and occupying land on the environment for human purposes, including the direct use of land and all indirect contributions related to the product (i.e., fuels, chemicals, feed, and fertilizers) that require land for its production. Carbon sequestration capacity was also derived from the scientific literature. The actual role of shellfish in carbon sequestration has been somewhat debated since the early 2000s, but most of the recent scientific literature is clearly oriented toward considering the shellfish production process as net carbon sequestration [47,48,49,50,51].

## 3. Results

### 3.1. Worldwide Protein Consumption by Source

**Figure 1:**
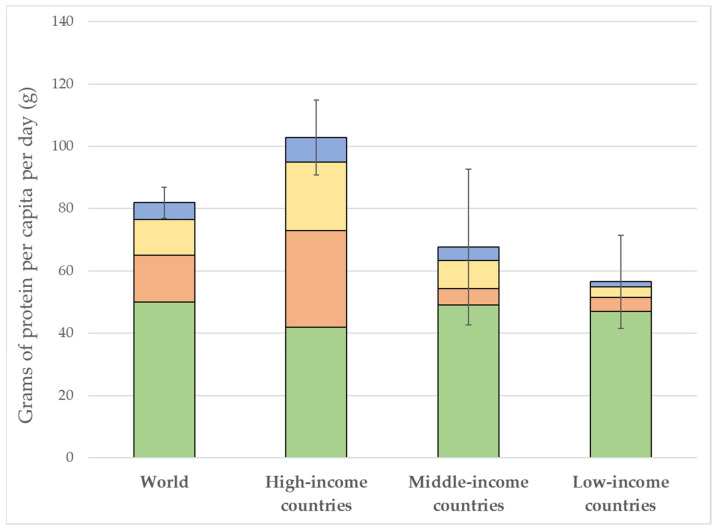
Protein consumption expressed in grams per capita per day by source: plant proteins (green bars), terrestrial animal meat (orange bars), eggs and diary proteins (yellow bars), and seafood (blue bars) (source FAO [23]; reference year, 2020). Based on the World Bank classification, countries have been grouped into high-income (with a gross national income per capita (GNI) above USD 12,375); middle-income (with a GNI between USD 1036 and USD 12,375); and low-income (a GNI of USD 1035 or less).

Using the World Bank’s classification of countries by income levels [76], Figure 1 depicts the worldwide average daily protein supply per capita. Vegetable protein sources continue to dominate protein consumption (about 60%), with meat providing about 15% of available proteins worldwide, although its contribution to the diet varies widely among countries [77,78,79].

In countries with a high standard of living, vegetables are reduced approximately to 35% of daily protein, eggs and dairy products contribute to 28%, and animal protein (terrestrial and seafood) cover up to 40% of the overall intake. From low-income to low-middle-income countries, the amount of animal protein rises, gradually closing the gap with high-income countries [80], confirming the strong correlation between per capita income and meat consumption. Seafood currently provides approximately 7% of total proteins consumed by the global population, including capture fisheries, and aquaculture [81].

Although global protein production has dramatically increased significantly since 1990 (Table 1), by approximately 60% for pork and beef and by more than 200% for fish and plants, the forecast for 2050 indicates only a modest increase for all sources, except wheat. In contrast, alternative proteins, like mycoprotein, insect protein, cultured meat, and, as we shall see, potentially shellfish protein, have tremendous growth potential for the next few decades.

In 2030, the per capita consumption of insects and insect-based products (meals and powders) is expected to grow by about 10,000% worldwide, reaching about 3 mln tons in 2030, whereas their consumption as a source of nutrition is almost exclusively restricted to Africa, South America, and South-East Asia at present [82]. Recent estimates indicate that over 2000 insect species are now considered edible, primarily crickets, mealworms, beetles, and caterpillars [83]. Similarly, cultured meat is now at an early stage of development, but it is expected to reach 0.4–2.1 mtons of production by 2030 and gradually replace approximately 30–40% of traditional meat consumption [84].

As shown in Table 1, all animal sources have similar protein content but very different by-products: edible weight ratios (from 29% of poultry to 66% of beef). Beef also has the worst FCR (10%) and the lowest feed nutritional score, expressed as NRF_9.3_ = 3.7. Meanwhile, insects and cultured meat have the highest FCR and zero waste but a lower nutritional score than plant sources.

The dietary protein content of raw, unprocessed beef, pork, and poultry products examined in this review ranged from 20.4 ± 1.7 g/100 g to 22.0 ± 3.6 g/100 g. Similarly, raw farmed fish products exhibited comparable protein levels, with rainbow trout providing 19.4 ± 1.7 g/100 g and salmon offering 21.2 ± 2.0 g/100 g. Insects (16.9 ± 3.9 g/100 g) and cultured meat demonstrated protein contents nearly equivalent to that of conventional meat and seafood products. In contrast, plant-based sources displayed significantly lower protein concentrations, ranging from peas (5.9 ± 1.2 g/100 g) to soy and wheat flour (14.3 ± 4.4 g/100 g and 14.0 ± 1.0 g/100 g, respectively). Moreover, from a nutritional standpoint, plant proteins often struggle to provide the diverse range of essential amino acids found in meat products [85]. Shellfish have a protein content comparable with that of plant proteins, the highest values of by-products (primarily shells), and the highest NRF_9.3_ compared with other proteins. A higher NRF_9.3_ corresponds to low energy-dense and healthier foods. Shellfish and seafood are lower in saturated fat than red meat, pork, and poultry, but they may not contain as many mineral nutrients as red meat, like iron and zinc. The unique dietary value of molluscan shellfish is them being low in calories but high in nutrients, especially minerals and vitamins that are not always found in other meats [86]. Moreover, they are rich in non-cholesterol sterols, low in saturated fatty acids, and rich in omega-3-fatty acids, which are essential for maintaining overall human health from reducing blood pressure to lowering risks of depression and anxiety [87]. In terms of efficiency, seafood species are very efficient converters of feed into protein, far more efficient than most terrestrial livestock systems. For instance, poultry converts about 18% of their consumed food and pigs convert about 13%, as compared with 30% in the case of fish [88] and up to 50% for shellfish [62]. As an example, based on the NRF_9.3_ values and average protein content of meats, it is estimated that 100 g of beef is required to provide the same nutritional score as 21 g of oyster meat, 13 g of mussel meat, or 12.5 g of clam meat.

### 3.2. Carbon Footprint of Protein Sources and Land Use

According to the selected LCA studies, referring to 1 kg of live/fresh weight and cradle-to-gate boundaries, the carbon footprint and land use of the different protein sources are presented in Table 2, which indicates that beef has the largest impact, both in terms of carbon dioxide equivalent (CO_2_ eq) emissions per 1 kg of live weight (24.5–42.6 CO_2_ eq/kg live weight) due to rumen fermentation and land use (43.5–420 m^2^/a·kg). Regardless of production systems, which range from highly intensive to highly extensive, or the choice between localized organic supply chains and global distribution, beef production requires substantial land use. Some studies estimate land consumption at up to 420 m^2^ per year per kilogram of live beef [89]. Additionally, it has been suggested that producing feed for Europe’s livestock demands cropland equivalent to seven times the size of the European Union [90]. Our data demonstrate a wide range of product variation. Pork and poultry have a medium-to-high carbon footprint and land consumption (4.8–11.0 CO_2_ eq/kg live weight and 8–15 m^2^/a·kg, respectively). In both cases, the variety may be the result of the availability of conventional production systems or free-range animals, which significantly impact both categories. For pigs, a significant portion of the emissions originate from nitrous oxide emissions released during feed production [91]. For farmed seafood, the CO_2_ eq. values are primarily driven by aquaculture methods and equipment and the distance to aquaculture sites, which in turn influence fuel use and overall energy consumption.

The allocation of terrestrial land for seafood production has been quantified as agricultural land utilized for cultivating the plant-based components of feed.

Intensive aquaculture practices for farmed salmon, rainbow trout, and milkfish require high-protein nutrients, additives, antibiotics, and drugs to improve the health and yield of farmed fish and prevent disease [92]. The production and use of these factors have a relatively small impact on the carbon footprint of seafood products but a significant impact on other impact categories of the LCA analysis, like ecosystem toxicity and human toxicity, that were not considered in this study. Plant products have a lower carbon footprint than animal proteins, as expected. Meanwhile, agricultural practices, such as the use of machinery and equipment and the production and use of fertilizers and pesticides, contribute significantly to their environmental impact [93]. According to Nemecek et al. [94], the land use parameter for agricultural products in LCA studies includes not only land use but also all contributions indirectly associated with cultivation. Cultivating bivalve mollusks as feed for farmed finfish could counteract all of the negative aspects of current practices in aquaculture feed production [95].

Although the uncertainty ranges are still large, some early studies have reported that in vitro biomass cultivation may require fewer inputs and land use than livestock production [96]. The impacts of carbon footprint are due to biomass cultivation, bioreactor cleaning, and facilities, whereas the contribution to land use is due to the use of starch and soy hydrolysate, which are considered the main ingredients for the production of cultured meat [97]. Similarly, for insects, LCAs have only been released for mealworms, house crickets, black soldier flies, and houseflies [98]. Feed production is again the main driver of carbon footprints and land use. Total CO_2_ eq. emissions are lower than conventional livestock’s, but energy requirements are still high because insects require relatively high temperatures during rearing [99].

Specifically, shellfish have the lowest carbon footprint [75]. The fact that they are filter feeders contributes to their relatively low environmental impact. The main contributors to CO_2_ eq emissions are plastic materials for farming and harvesting, fuel consumption, boat use and maintenance, and, in the case of mussels and oysters, farming facilities, such as off-shore long-line farms [100]. Bivalve mollusks also have a negligible land use contribution, because they occupy land only for land-based building and ancillary facilities and do not need soil to produce external feed [19]. Moreover, their contribution to the occupation of a marine area is also negligible, since it has been calculated in the range 1–2 m^2^/a crop equivalent per kilograms of farmed bivalves [74]. Thus, bivalve aquaculture is generally characterized by a low energy input and established technologies, which lead to a low carbon footprint related to production [101,102]. A more detailed analysis of the carbon footprint methods and calculations related to the protein source production mentioned in this study can be found in the cited literature.

### 3.3. Carbon Capture Capacity

Agriculture is not only a major source of carbon but could also be a sink due to the functions of soils and crops [103]. Green plants absorb CO_2_ and fix it in organic substances that can be retained in the soil or released back into the atmosphere through photosynthesis, which brings carbon into terrestrial ecosystems, including agriculture. The factors that influence carbon sources and sinks in agroecosystems are highly heterogeneous and complex, depending on climate patterns, soil types and properties, and management practices [104]. The issue of carbon sequestration and reduction through agriculture has received considerable attention, and it is beyond the scope of this study to look at it. Here, we limit ourselves to pointing out that crops can contribute to carbon storage in terrestrial ecosystems. Peas have a significant carbon sequestration capacity, similar to the amount of CO_2_ eq. emitted for their production. The wide range of wheat depends on whether it is considered a winter cover crop [105,106]. Furthermore, photosynthetic organisms are only net carbon absorbers when photosynthesizing. At night, plants emit respiratory CO_2_ like every other aerobic organism, and when the plant dies, carbon sequestered in its biomass is digested by a multitude of bacteria, fungi, and detritivores that release all the biomass carbon back to the atmosphere as respiratory CO_2_ [107].

Terrestrial animals and seafood, as well as insects, are emitting organisms, both in terms of physiological metabolism and production process; hence, their contribution to this parameter is more doubtful. Similarly, there is no point in defining the parameter for cultured meat. On the contrary, due to the natural biocalcification process that leads to calcium carbonate formation, bivalves are the only animal protein source that have the potential to act as a carbon sink during life, leaving a legacy of deposited limestone when they die. In fact, their shell is principally composed of mineral calcium carbonate (CaCO_3_) formed during a biological process called biogenic calcification or biomineralization. The stoichiometry of the chemical reaction that occurs during biocalcification provides that for every two CO_2_ molecules captured from seawater, one is returned to the marine carbon cycle, and one is permanently stored in the shell as insoluble CaCO_3_ [108]. Bivalve mollusks are farmed for harvest and human consumption; similarly, shells are permanently removed from the water at the end of the production cycle, resulting in net sequestration. Clams and oysters have a high carbon sequestration capacity due to their higher shell-to-flesh ratio than mussels (about 60% for clams and 75% for oysters vs. 35% of mussels).

## 4. Discussion

Based on the environmental Kuznets curve [109], developed countries show greater environmental awareness, while people in developing countries still aspire to Western consumption patterns. Bennett’s law demonstrates that rising incomes lead to shifts from starchy staples toward refined grains, fruits, vegetables, meat, and dairy products [110]. These models explain the projected increase in livestock product demand by mid-century. Managing this requires modeling sustainable protein sources for human consumption to avoid exceeding planetary boundaries. Adequate dietary protein is essential for meeting the body’s indispensable amino acid requirements, including phenylalanine, valine, threonine, tryptophan, isoleucine, methionine, histidine, leucine, and lysine [111]. Animal-derived proteins like meat, seafood, eggs, and dairy are considered “high-quality” due to their essential amino acid content [112], while plant-based proteins often contain some essential amino acids but are limited in others [113]. The issue of global protein supply extends beyond substituting animal proteins with plant sources. While this might reduce immediate impact, increased plant protein production requires new agricultural land, which is scarce and would impact forests. Intensive agriculture has become unsustainable due to fertilizer misuse. In 2018, synthetic nitrogen fertilizers generated 1.13 Gt CO_2_ eq. emissions, comprising 10.6% of agricultural and 2.1% of global greenhouse emissions [114]. Expanding arable land increases CO_2_ emissions and reduces biodiversity. While pulses and cereals can supplement animal proteins, they cannot fully replace them, as plant proteins alone are incomplete in amino acid composition for human health despite their vitamins and fiber content [115].

Insect production is touted as the future, offering low environmental impacts, land requirements, and feed/water inputs [116]. However, problems exist like high energy needs for heating, pest control, and maintaining hygienic conditions. Cultural barriers in Western societies make large-scale adoption difficult, except as hidden ingredients. Cultured meat allows for controlling the nutritional content by adjusting the production medium, such as increasing polyunsaturated fatty acids or micronutrients [117]. While requiring less land than livestock, its environmental benefits remain controversial. Beyond high costs, consumer acceptance and ethical–religious concerns need consideration when comparing current meat production. The production of farmed finfish and crustaceans has grown exponentially, severely impacting marine ecosystems through feed and chemical use [118]. Plant-derived feed affects terrestrial land use, while seafood fed with wild-caught fish worsens marine resource overexploitation [119]. Factory ships can destroy indigenous fishing industries. Aquaculture effluents containing fertilizers, drugs, and additives contribute to pollution and ecosystem toxicity in coastal regions. The high nutritional value of seafood and crustaceans makes them vital for human diets. Improving FCR and selecting appropriate feeds could reduce environmental impacts [120].

The data show the inadequacy of protein supply solutions ensuring food security and environmental protection. Bivalves offer a promising solution, though cultural and economic factors hinder widespread adoption. Limited knowledge, funding, infrastructure, and consumer acceptance remain barriers to expanding bivalve aquaculture. However, over 1,500,000 km^2^ of undeveloped coastline is suitable for bivalve farming [121]. China’s success since the 1950s, producing 85% of the global supply, demonstrates bivalve aquaculture’s viability [122]. Expanding this model across Asia, Africa, the Americas, and Oceania could benefit health and sustainability.

According to Morales-Nin et al. [123], bivalve production’s carbon footprint is 20 times lower than that of beef and 10 times lower than that of chicken and pork per serving. Substituting 10% of beef or chicken consumption with oysters could avoid 360 million and 120 million tons of CO_2_-equivalent emissions annually. Carbon sequestration in shells could reduce atmospheric CO_2_ by 2–3 mtons yearly, requiring 1.4–2.2 million hectares using terrestrial carbon capture systems [47]. While bivalves’ carbon seizure is small compared to global emissions, it provides permanent sequestration, unlike reforestation or conservative agriculture [124]. Even advanced engineering solutions have not proven long-term storage capability, yet fossiliferous limestone strata were produced by calcifying organisms millions of years ago [125].

The key point worth highlighting is that the carbon sequestration capacity of shellfish can be considered a side-benefit at zero cost because it occurs while the mollusk is being cultivated to provide human food. Bivalve aquaculture could have an effective and significant role in fighting against climate change, especially in coastal countries, and help reach Goal #13 without creating environmental and economic issues. As global food demand continues to rise in the coming decades, aquaculture is likely to expand, driven by its human health benefits and increasing consumer preference for seafood [126]. Bivalve aquaculture has a minimal contribution to food production-related emissions and can be further enhanced through responsible practices. Ultimately, the advancement of aquaculture is a crucial strategy for meeting growing food demands while ensuring food security within planetary boundaries [127]. Recent estimates indicate that emissions from bivalve production account for only 7.6% of the average emissions associated with terrestrial protein sources [19]. As a result, bivalve aquaculture is now widely recognized as a sustainable, climate-friendly means of producing nutrient-rich protein for human consumption. Additionally, beyond its role in food production, bivalve farming contributes as a net carbon sink, with current Manila clam production alone sequestering approximately 1 million tons of carbon per year, in support of global efforts toward UN Sustainable Development Goal #13.

A significant issue with bivalve consumption, particularly oysters, is the waste generated relative to the meat yield. Several options for waste shell valorization have been reported, showing increasing interest [113]. One possibility for using bivalve shells, combining carbon sequestration with ecosystem benefits, is creating coastal defense structures and submerged reefs [128]. The use of shells would promote bivalve larvae settlement, with additional carbon storage potential [129].

## 5. Conclusions

The growing need to ensure food security for an expanding global population, coupled with promoting the sustainable use of natural resources, has heightened interest in bivalve aquaculture. Unlike intensive fish farming, bivalve aquaculture is a zero-emission system that relies on natural phytoplankton, requiring minimal intervention with no additives or antibiotics that could affect its carbon footprint. The production of bivalves plays a significant economic role in coastal regions worldwide, particularly in developing countries. Notably, bivalve farming is the only food production method maintaining a positive net carbon balance in terms of sequestration capacity. Given the global demand for nutrient-rich food sources that do not strain environmental resources, coastal areas remain available for sustainable development. However, challenges must be addressed to unlock bivalve aquaculture’s full potential. Harnessing innovation and technologies can help overcome these challenges, expanding bivalve aquaculture as a viable economic activity that provides sustainable income while feeding nearly a billion people in developing regions. By linking bivalve farming to environmental goals, this study supports climate-friendly aquaculture practices that generate ecological and economic benefits. Findings show that bivalve farming offers advantages in carbon sequestration and plays a crucial role in coastal marine ecosystems’ carbon cycles.

Bivalves aquaculture could help achieve 5 of the 17 UN’s SDGs of Agenda 2030: (1) SDG Goal #1 (no poverty), as sustainable bivalves aquaculture creates income opportunities for coastal communities; (2) SDG Goal #2 (zero hunger), as sustainable aquaculture ensures food security; (3) SDG Goal #3 (promoting health and wellbeing), due to nutritional benefits; (4) SDG Goal #12 (responsible consumption, and production), as it follows natural growing cycles without antibiotics and has lower environmental impacts than other foods; and (5) SDG Goal #14 (life below water), as bivalves aquaculture can reduce pressure on marine ecosystems while providing environmental benefits.

## Figures and Tables

**Table 1:** Production, consumption, and characterization of the principal proteins by source (FAO, 2020; [23]).

Protein Source	World Production (2020)	World Production Increasing Since 1990	World per Capita Consumption (2020)	World per Capita Consumption Increase by 2050	Protein Content per 100 g of Live or Raw Product	By-Products/Edible Weight	FCR	NRF_9.3_	Source
	(Mtons * of live ** or fresh *** weight)	(%)	(g/day)	(%)	(g)	(%)	(kg feed: kg live or fresh weight)		
**Poultry**	133.4	260	109.2	66	20.9 ± 2.9	29	2.5:1	10.4	[24,27,28,29,30]
**Pork**	109.8	60	112.3	28	22.0 ± 3.6	52	5.0:1	7.0	[24,27,28,30,31]
**Beef**	72.1	56	68.0	56	20.4 ± 1.7	66	10:1	3.7	[24,27,28,30,32]
**Whole wheat flour**	752.0	200	180.0	395	14.0 ± 1.0	32	-	32.3	[24,30,33,34]
**Soy**	353.5	220	123	186	14.3 ± 4.4	22	-	21.0	[24,30,35,36]
**Pea**	14.6	0	9.3	NA	5.9 ± 1.2	30	-	23.1	[24,30,37,38]
**Farmed salmon**	2.7	200	35	4.3	21.2 ± 2.0	38	1.35:1	21.8	[24,30,38,39]
**Farmed milkfish**	1.3	200	NA	NA	21.0 ± 2.3	45	1.35:1	21.8	[24,26,30,40,41]
**Farmed rainbow trout**	0.8	170	25	NA	19.4 ± 1.7	45	1.35:1	21.8	[24,26,30,42,43]
**Farmed crustacean**	9.4	370	7.2	200	21.7 ± 2.5	62	2.4:1	3.6	[24,26,30,40]
**Insect**	0.2	0	NA	10,000	16.9 ± 3.9	0	1.2–2.2:1	2.15–13.14	[28,44,45]
**Cultured meat**	0.0	0	NA	NA	av. 20% < meat	0	1.2:1	NA	[32,46]
**Clam**	4.4	800	19.8	44.5	12.8 ± 2.7	56	-	46.9	[24,30,32]
**Mussel**	1.1	16			11.9 ± 3.1	35	-		
**Oyster**	6.0	400			7.5 ± 1.9	90	-		

* Mtons = 10^6^ tons. ** live weight = the weight of an animal before it has been slaughtered and prepared as a carcass. *** fresh weight = the weight of vegetable food recently harvested or obtained from the plant, not preserved, is in its raw, unprocessed state.

**Table 2:** Cradle-to-gate carbon footprint (expressed as kg CO_2_ eq. per kg of live weight or fresh product), land use (measured as m^2^ of agricultural land per year and per kg of product), and carbon sequestration capacity for the protein sources selected for the present study.

Protein Source	Carbon Footprint (kg CO_2_ eq. */kg live ** or Fresh *** Weight)	Land-Use (m^2^/a·kg) ****	Carbon Sequestration Capacity (kg CO_2_ */kg live ** or Fresh *** Weight)	Source
**Poultry**	2.0–6.8	5–8	-	[52,53]
**Pork**	4.8–11.0	8–15	-	[52]
**Beef**	24.5–42.6	43.5–420	-	[54,55,56]
**Whole wheat**	1.2–1.5	0.9–3.8	0.03–0.32	[57,58,59]
**Soy**	0.7–0.8	2.8–6.8	0.10–0.14	[60,61]
**Pea**	0.5	3.2–4.9	0.12–0.55	[62]
**Farmed salmon**	3.8–16.7	3.1	-	[63]
**Farmed milkfish**	3.6–4.7	2.6–4.1	-	[64,65,66]
**Farmed rainbow trout**	2.2–3.7	0.8–1.6	-	[66,67]
**Farmed crustaceans**	3.1–6.8	2.2	-	[66,68,69]
**Insect**	2.7–4.0	3.6	-	[70,71]
**Cultured meat**	6.6–25	7.7	-	[72]
**Clam**	0.02–0.7	5.0 × 10^−5^	0.15–0.27	[47,73,74,75]
**Mussel**				
**Oyster**				

* kg CO_2_ eq. = “kilogram of carbon dioxide equivalent” is a unit of measurement used to compare the global warming impact of different greenhouse gases, expressing them in terms of carbon dioxide. ** live weight = the weight of an animal before it has been slaughtered and prepared as a carcass. *** fresh weight = the weight of vegetable food recently harvested or obtained from the plant, not preserved, is in its raw, unprocessed state. **** (m^2^/a·kg) = “square meters per year per kilogram” is a unit of measurement used to quantify the amount of land area (measured in square meters) required to produce a certain amount of a product (measured in kilograms) over a specific time period (measured in years).

## Data Availability

The original contributions presented in the study are included in the article. Further inquiries can be directed to the corresponding author.
